# Immune Modulation in Critically Ill Septic Patients

**DOI:** 10.3390/medicina57060552

**Published:** 2021-05-31

**Authors:** Salvatore Lucio Cutuli, Simone Carelli, Domenico Luca Grieco, Gennaro De Pascale

**Affiliations:** 1 Dipartimento di Scienze dell’ Emergenza, Anestesiologiche e della Rianimazione, Fondazione Policlinico Universitario A. Gemelli IRCCS, 00168 Rome, Italy; simonecarelli.sc@gmail.com (S.C.); dlgrieco@outlook.it (D.L.G.); gennaro.depascalemd@gmail.com (G.D.P.); 2 Facoltà di Medicina e Chirurgia “A. Gemelli”, Università Cattolica del Sacro Cuore, 00168 Rome, Italy

**Keywords:** sepsis, septic shock, infection, extracorporeal immune modulation, blood purification, renal replacement therapy

## Abstract

Sepsis is triggered by infection-induced immune alteration and may be theoretically improved by pharmacological and extracorporeal immune modulating therapies. Pharmacological immune modulation may have long lasting clinical effects, that may even worsen patient-related outcomes. On the other hand, extracorporeal immune modulation allows short-term removal of inflammatory mediators from the bloodstream. Although such therapies have been widely used in clinical practice, the role of immune modulation in critically ill septic patients remains unclear and little evidence supports the role of immune modulation in this clinical context. Accordingly, further research should be carried out by an evidence-based and personalized approach in order to improve the management of critically ill septic patients.

## 1. Introduction

Sepsis [1] represents an acute syndrome of major interest for intensive care physicians because of significant incidence and severe clinical outcomes [2]. Pathophysiology of sepsis originates from a non-physiological, non-protective, non-adaptive inflammatory response to microbiological threats [1]. Identification and control of the source of infection [2] as well as timely and appropriate antibiotic therapy [3] were shown as the most effective interventions that may improve sepsis-induced organ dysfunction. Accordingly, a pathophysiological approach to sepsis is strongly advocated. In the light of this view, immune modulation by pharmacological and extracorporeal blood purification therapies (EBPT) represents a complementary therapy for sepsis and many studies have been conducted with the aim to find a role for such an intervention in this field. In this paper, we clarified the rationale and the role of immune modulation in critically ill septic patients.

## 2. Immune Alteration in Sepsis

### 2.1. Pathophysiology of Immune Alteration in Sepsis

Sepsis is a life-threatening organ dysfunction, which is caused by dysregulated host response to infection [1]. Sepsis is an old disease [4] and seminal research hypothesized a causative link between the pathogenicity of specific microorganisms and the severity of this syndrome. However, recent research, most of which was based on molecular assessment of human inflammatory genes, has described the pivotal role of host response in the development of sepsis-associated organ dysfunction and consequent clinical outcomes [5,6]. Specifically, sepsis results from host-pathogen interactions that occur when microorganisms invade sterile organs of the body as well as when microbiota are altered by concurrent conditions (e.g., drug and diet) that shift symbiosis to dysbiosis [7,8]. In some patients, this process results in an exaggerated, uncontrolled, and self-sustaining systemic inflammatory response that causes metabolic derangements and organ dysfunction [6].

Immune response to pathogen invasion is initiated by the recognition of highly conserved pathogen-associated molecular patterns (PAMPs) and danger-associated molecular patterns (DAMPs), which belong to microorganisms and injured tissues of the host, respectively. These molecules are recognized by specific receptors (e.g., Toll-like Receptors) that activate multiple intracellular pathways. Specifically, the activation of selective receptors induces the phosphorylation of mitogen-activated protein kinases (MAPKs), Janus kinases (JAKs), or signal transducers and activators of transcription (STATs) [9]. These molecular pathways induce the expression of specific genes, which codify for inflammatory (e.g., cytokines) and metabolic molecules (e.g., hormones) that orient host response to deal with microbial threats. Moreover, PAMPs e DAMPs trigger further cellular (e.g., neutrophil release of toxic agent) and non-cellular (e.g., complement activation) responses that magnify immune response to pathogen invasion [10]. Among PAMPs, lipopolysaccharide (LPS), a molecule of the outer membrane of the Gram negative bacteria, has been found to induce a dose-dependent activation of the inflammatory system [11]. Among DAMPs, nuclear and cytosolic factors as well as hyaluronan and heparan sulfate of the extracellular matrix are potent activators of the immune system response [12]. On the other hand, a growing body of evidence supports the role of microbiota as organs that may influence immune system response to infection and induce tolerance towards specific molecules (e.g., endotoxins) [13,14,15], which may have an impact on patient-related clinical outcomes.

The physiological inflammatory response to pathogen invasion of the body implies immune activation and immune suppression, while sepsis occurs when the balance between these pathways is lost [9]. Traditionally, immune activation was considered as the early stage of inflammation, which is triggered by innate pathways of response. Many cytokines have been identified as immune-activating molecules and include tumor necrosis factor-α (TNF-α), several interleukins (e.g., IL-1β, IL-2, IL-6, IL-8), and interferon-γ (IFN-γ). On the other hand, immune suppression was considered the late stage of inflammation, which was intended to extinguish immune activation when the pathogen threat is solved. This stage is mediated by the release of specific molecules like IL-10 and is pathologically exaggerated when chronic critical illness occurs [16].

### 2.2. Immune Alteration-Induced Organ Dysfunction in Sepsis

In the last few years, an increasing body of evidence has demonstrated that immune activation and immune suppression happen concurrently and cause organ dysfunction, and the severity of which may be evaluated by the Sequential Organ Failure Assessment (SOFA) score [17] (Table 1). The SOFA score has been demonstrated important to synthetize and report sepsis-associated organ dysfunction as well as to provide prognostication for this patient population [18]. Moreover, a simplified version of the SOFA score, namely the quick SOFA (qSOFA) [1], has been identified as an effective tool to identify patients with suspected infection outside the ICU, at risk of poor clinical outcomes. The qSOFA has such an important diagnostic implication when at least two of the following clinical criteria are present: respiratory rate of 22/minute or greater, altered mentation and systolic blood pressure of 100 mmHg or less [19].

## 3. Immune Modulation in Sepsis

### 3.1. Rationale of Immune Modulation in Sepsis

Immune alteration represents the main pathological pathway that causes and sustains sepsis. Accordingly, immune modulation has appeared as a promising adjuvant therapy in patients who suffer from such disease. Immune modulation may be carried out by specific interventions with the aim to mitigate both pro- and anti-inflammatory bursts, thus allowing for an appropriate and protective response to microbial threat. Immune modulation should be considered as a complementary therapy and should be used with the aim of limiting infection-induced inflammatory alteration by the time appropriate etiologic therapies (e.g., source infection control and antibiotics) are delivered to the patient [2].

### 3.2. Indirect Immune Modulation in Sepsis

In order to limit immune alteration caused by host response to infection, the microbiological threat must be identified and treated. Such an approach implies the identification of both source (organ or system) and agent (bacterium, virus, parasite or fungus) that cause infection. The source of infection must be determined by clinical assessment (e.g., symptoms) of the patient and possibly confirmed by radiological examination (e.g., Ultra-Sound Scan, chest X-Ray, or CT-scan) [2]. The identification of the source of infection may guide the decision to withdraw samples from specific organs (e.g., cerebrospinal fluid from the central nervous system) that will be tested to identify the agent responsible for infection. In this context, blood samples should always be withdrawn and sent for microbiological examination in order to identify systemic diffusion of the microorganism, which may be associated with the risk of delivering infection to other sites [2]. The identification of the microbiological threats offers the possibility to target antimicrobial therapy to the etiologic cause of infection and deliver an appropriate treatment [2]. Moreover, identifying the source offers the possibility to control the progression of infection at a local level by surgery (e.g., intestinal resection after organ perforation) or interventional radiology (e.g., drainage of an abscess) [2].

### 3.3. Direct Immune Modulation in Sepsis

#### 3.3.1. Pharmacologic Immune Modulation in Sepsis

Many different drugs have been tested with the aim to provide immune modulation in patients with sepsis (Table 2).

The pathophysiological hypothesis beyond the administration of immune modulating drugs in patients with sepsis relies on the concept of smoothing both hyper- and hypo-inflammation via synthetic analogues of cytokines that are intended to hold such features. As an example, IFN-γ and the granulocyte-macrophage colony-stimulating factor (GM-CSF) have been investigated in order to provide immune modulation due to pleiotropic effects on innate inflammation. The administration of these drugs has shown controversial efficacy and no significant adverse events [20,21]. However, the administration of these drugs was conducted under specific clinical criteria that did not take into account any immune system biomarker (C-reactive protein, cytokines), which may have hampered the results of trials. Specifically, GM-CSF has been demonstrated as effective to improve immune suppression in other clinical contexts and provide some benefit on attenuating lung remodeling in patients with pulmonary fibrosis [22] or immunosuppressive T-regulatory cells replication in cancer vaccine therapy [23]. Moreover, the administration of cytokine analogues like IL-7 have shown significant anti-apoptotic and lymphopoietic effects on T-cells, which may reverse sepsis-associated lymphocyte depletion. Recombinant IL-7 has been described to improve survival in animal models of bacterial and fungal sepsis [24,25], although no definitive clinical evidence supports its use in daily clinical practice.

Recently, complement manipulation may play a role in the development of sepsis-associated immune alteration. Specifically, C5a activity has been demonstrated as crucial in the development of inflammatory mediated tissue damage and its inhibition via selective antibodies was demonstrated effective to mitigate sepsis severity in animal models [26]. However, no definitive clinical data support the use of this therapy in daily clinical life. On top of that, an increasing amount of evidence has shown the interaction between complement and coagulative systems [27]. The latter is frequently altered in patients with sepsis and many drugs have been tested with the aim to improve coagulative dysfunction. However, the administration of recombinant human soluble thrombomodulin [28] as well as activated protein C [29] did not show any benefit on 28-day mortality of critically ill patients with sepsis.

In the last decades, the administration of intravenous immunoglobulins (IVIg) has been increasing in patients with sepsis and such therapy appears characterized by multiple mechanisms of action that include pathogen recognition and killing, toxin scavenging, inflammatory genes-reduced transcription, and anti-apoptosis effects on immune cells [30]. Both polyclonal and monoclonal IgG as well as IgM-enriched polyclonal antibodies have been tested as adjuvant therapies. However, no significant benefits on patient-related outcomes have been observed in clinical trials [31]. As a result, current guidelines [2] do not recommend the use of IVIg in patients with sepsis. On the other hand, small sample sizes and the heterogeneity of IVIg formulations tested in clinical trials support the need for further investigations on the role of this adjuvant therapy in patients with sepsis [32].

Moreover, glucocorticoids are drugs with immune-modulating properties and mimic hormones that are released by adrenal glands when the organism in under stress [33]. Glucocorticoids exert long lasting immune suppressing effects by inhibiting cellular synthesis of pro-inflammatory cytokines [34]. Although the administration of Dexamethasone and Methylprednisolone may increase the risk of secondary infections [35] in patients with sepsis, Hydrocortisone appeared safe and effective to shorten shock duration, mechanical ventilation and ICU length of stay [36]. On the contrary, Methylprednisolone decreased treatment failure of patients with severe community-acquired pneumonia and high initial inflammatory response [37] while Dexamethasone was demonstrated effective to reduce 28-day mortality of patients with acute respiratory failure caused by Coronavirus Disease 19 (COVID-19) [38]. As a result, Hydrocortisone is recommended in patients with septic shock [2], Dexamethasone in patients with COVID-19, and Methylprednisolone, as a rescue therapy, in patients with severe community-acquired pneumonia [39].

Finally, many drugs have been tested with the aim to provide immune modulation via the interaction with ultra-specific pathways of inflammatory host response to infection. As an example, the administration of Sivelestat, a neutrophil elastase inhibitor, may play some role to improve the outcome of septic patients with acute respiratory distress syndrome and disseminated intravascular coagulation [40]. Moreover, sepsis-associated immune paralysis may be improved by the administration of immune checkpoints such as the programmed cell death protein 1/programmed death ligand (PD-1/PD-L) pathway inhibitor [41]. Furthermore, Heme oxygenase inducers promote oxidative conversion of Heme to carbon monoxide, iron, and biliverdin, thus playing pleiotropic modulation of inflammatory pathways involved in host response to infection [42]. In summary, neutrophil elastase inhibitors, PD-1/PD-L, and Heme oxygenase inducers represent promising immune modulating therapies in critically ill septic patients and ongoing clinical trials will shed light on their role in this population.

#### 3.3.2. Extracorporeal Immune Modulation in Sepsis

Extracorporeal removal of PAMPs, DAMPs, and cytokines is considered the new frontier of immune modulation in patients with sepsis. Such interventions allow mediators removal from the bloodstream via specific characteristics of the internal surface of membranes. Moreover, their application in critically ill patients with sepsis appeared feasible and was made easy by the significant rate of acute kidney injury that required continuous renal replacement therapy (CRRT) [43]. Accordingly, EBPT allows selective and non-selective removal of mediators, thus providing short term immune modulation and preventing long-term immune complications that were associated with longer-lasting pharmacological interventions. In the light of this view, the last version of the Surviving Sepsis Campaign Guidelines [2] refers to EBPT as complementary treatments that should be applied with the aim to provide immune system control and multi-organ support by the time etiologic treatments will be delivered to the patient (e.g., control of source of infection and antibiotics).

EBPT are characterized by important features that should be considered when prescribing such interventions [44]. First, each device is characterized by a certain degree of biocompatibility, which refers to the level of complement and platelet activation that results from the interaction between blood and artificial surfaces [44]. Biocompatibility may influence the half-life of the device, condition its efficacy, and worsen inflammatory burst of the host. Although any device available in the market must adhere to specific requirements of the ISO10993, no clinical data exist on the comparison of different devices in terms of biocompatibility [44]. Moreover, EBPT may cause unintended removal of drugs or vitamins, which may have a non-favorable impact on patients’ related clinical outcomes. Specifically, lowering antibiotic blood concentration by extracorporeal removal may worsen infection control and increase sepsis-associated inflammatory burst with consequent life-threatening complications [44]. Accordingly, antibiotic dosage should be adapted to any specific EBPT and a strict control of antibiotic blood level concentration is strongly advocated due to the lack of information about clearance characteristics of the majority of new membranes available in the market [44]. Third, EBPT imply a certain degree of heat dissipation to the environment, despite any device for such therapy being endowed by heaters. Heat dissipation may mask fever and cause hypothermia, thus increasing peripheral vasoconstriction [45] and risk of organ hypoperfusion as well as conditioning drug solubility in the bloodstream, enzymes function, and mediators removal at a membrane level. Moreover, hypothermia itself was associated with increased organ dysfunction and 28-day and in-hospital mortality in critically ill patients [46].

Main Application of Extracorporeal Immune Modulation in Critically Ill Septic Patients

Mediators removal via extracorporeal therapy may be selective or non-selective [44] (Table 2). Selective removal of mediators is allowed by specific interaction between soluble molecules and membrane characteristics.

▪ Non selective extracorporeal removal of inflammatory mediators

PAMPs and cytokines may be non-selectively removed by EBPT via:

- electrostatic interactions between soluble molecules and the internal surface of the membrane (adsorption);
- trans-membrane flux via gradient (diffusion via hemodialysis) and pressure (convection via hemofiltration) concentration, according to the cut-off of the device.

Electrostatic interactions regulate mediator removal of many different devices for EBPT. Specifically, acrylonitrile-69 surface-treated (AN69-ST, Baxter, IL, USA) and surface modified membranes (Oxiris^®^, Baxter, IL, USA) are devices for CRRT that are characterized by heparin-coated polymers of sodium methallylsulfonate and polyethyleneimine. They allow adsorption of both pro- and anti-inflammatory cytokines (tumor necrosis factor α, IL 6, IL 8, and interferon γ) as well as endotoxin (Oxiris^®^), both in vitro [47] and in patients with septic acute renal failure [48]. Moreover, EBPT with Oxiris^®^ was associated with significant reduction of IL-6 blood level concentration in critically ill patients admitted to the ICU for COVID-19 [49,50].

Another EBPT which allows for CRRT and mediators removal by adsorption is Hemofeel^®^ (Toray Medical Co Ltd., Tokyo, Japan), a device made by polymethylmethacrylate that was demonstrated as effective in the removal of IL-8 and IL-6 by in-vitro study [51]. However, no clinical evidence exists on the effect of such therapy on the outcome of critically ill patients with sepsis.

Among EBPT that allow mediators removal via adsorption, Cytosorb^®^ represented a promising tool to deliver immune modulation in patients with sepsis. This cartridge is made by highly porous polystyrene divinylbenzene copolymer covered with a biocompatible polyvinylpyrrolidone coating and in-vitro studies demonstrated a certain degree of efficacy to remove pro- and anti-inflammatory cytokines [47]. However, a recently published randomized trial, which enrolled critically ill patients with sepsis, did not demonstrate any effect of Cytosorb^®^ hemoperfusion compared with standard care on IL-6 blood level concentration and 60-day mortality [52].

Moreover, the Seraph^®^-100 is a sorbent made by polyethylene beads, whose internal surface contains heparin. Although in vitro studies have shown some efficacy of this EBPT on cytokines (TNF-α), bacteria (Staphylococcus Aureus) and viruses (Zika virus, Cytomegalovirus, Adenovirus and Severe Acute Respiratory Syndrome Coronavirus-2) by adsorption [53], no clinical evidence exists on the effect of such therapy on the outcome of critically ill patients with sepsis.

On the other hand, Coupled Plasma Filtration and Adsorption (CPFA) represents a hybrid EBPT which allows mediator removal via plasma filtration and adsorption by styrene resin. Although in vitro studies demonstrated a direct relationship between cytokines removal and volume of plasma cleared by such device, a recent randomized controlled trial was stopped because of futility. Furthermore, this trial observed a significant rate of clotting (48% of the treatments) despite anticoagulation with heparin [54].

Finally, immune modulation may be performed by trans-membrane removal of mediators via gradient (diffusion via haemodialysis) and pressure (convection via hemofiltration) concentration. However, only membranes with a large pore size (20 nm) [55], namely high cut-off membrane (HCO), have been demonstrated as effective to remove inflammatory mediators (the majority of which have a molecular weight above 60 kDa). Although convection appears more effective than diffusion for mediator removal, the significant albumin loss associated with the former is of concern [56]. Accordingly, diffusive modalities are preferred when HCO membranes are used. Immune modulating effect of EBPT via HCO membranes have been suggested by an increasing number of randomized controlled trials that demonstrated significant cytokines blood level reduction when this therapy was compared to conventional renal replacement therapy [56,57,58,59,60]. Despite such promising effect of HCO EBPT on mediator removal, this intervention has not been demonstrated effective on other patients’ related clinical outcomes and its application in daily clinical practice is still a matter of debate.

▪ Selective extracorporeal removal of inflammatory mediators

To the best of our knowledge, endotoxin is the only PAMP that may be selectively removed via adsorption by Toraymyxin^®^ (Toray Industries, Tokyo, Japan) hemoperfusion. Toraymyxin^®^ is a cartridge made by polystyrene fibers and Polymyxin-B, a cationic antibiotic that is characterized by high affinity for endotoxin via ionic and hydrophobic bonds [47]. This device has been widely used in daily clinical practice [61,62], although randomized controlled trials carried out in this field have shown controversial results [63]. However, these trials enrolled patients with inhomogeneous characteristics mainly due to comorbidities, clinical severity, type of infection, timing, and protocol of EBPT provided that do not allow any final conclusion in this field. On the other hand, Toraymyxin^®^ was demonstrated as effective to improve the outcome patients at high risk of mortality (above 30%) [64] and for whom endotoxin level did not exceed the capability of the cartridge to remove such a molecule [65]. Moreover, Toraymyxin^®^ has shown immune modulating effect beyond endotoxin removal and very recently it was demonstrated effective as to improve immune suppression by allowing Monocyte Human Leukocyte Antigen-DR increase [66]. Finally, Toraymyxin^®^ hemoperfusion was used in a cohort of critically ill patients admitted to the ICU for COVID-19 [67] who developed secondary bacterial infections and for whom blood endotoxin activity was deemed implicated in the pathophysiology of immune system alteration and organ dysfunction.

### 3.4. Filling the Gap of Immune Modulation in Sepsis

Immune modulation offers enticing perspectives of treatment for critically ill septic patients. However, the real application of this complementary treatment is still a matter of debate due to controversial results between laboratory and clinical trials. Sepsis is a clinical syndrome, which complex pathophysiology may be explained by the multifaced genetic (e.g., polymorphic inflammatory pathways) and epigenetic (e.g., comorbidities and clinical intervention applied) interplay that characterizes each single patient. Accordingly, a personalized approach to sepsis may address such a gap via the clinical application of biomarkers of single-cell transcriptomics [68], big data analysis [69], and machine-learning methods by specific models [70], in order to identify specific patient populations that may benefit more from some specific immune modulating intervention and help the design of future clinical trials.

## 4. Conclusions

Immune modulation represents a complementary therapy for critically ill patients with sepsis. Among immune modulating strategies, EBPT appear safe and timely targeted compared with longer lasting pharmacological therapies. However, little evidence supports the efficacy of immune modulation in critically ill patients with sepsis. Accordingly, immune modulation remains a matter of debate and further research, carried out by evidence-based and personalized approaches, is warranted in order to improve the management of critically ill septic patients.

## Figures and Tables

**Table 1:** The Sequential Organ Failure Assessment (SOFA) Score.

Systems	Score				
	0	1	2	3	4
Respiration, PaO_2_/FiO_2_ ratio, mmHg (kPa)	≥400 (53.3)	<400 (53.3)	<300 (40)	<200 (26.7) with respiratory support	<100 (13.3) with respiratory support
Coagulation, Platelet count, cells × 10^3^/mm^3^	≥150	<150	<100	<50	<20
Hepatic, Bilirubin, mg/dL (μg/L)	≤1.2 (20)	1.2–1.9 (20–32)	2–5.9 (33–101)	6–11.9 (102–204)	≥12 (204)
Cardiovascular MAP, mmHg Catecholamines, μg/kg/min for at least 1 h.	≥70 -	<70 -	- Dopamine < 5 Dobutamine (any)	- Dopamine 5.1–15 or epinephrine ≤ 0.1 or norepinephrine ≤ 0.1	- Dopamine > 15 or epinephrine > 0.1 or norepinephrine > 0.1
Central Nervous System, Glasgow Coma Score	15	13–14	10–12	6–9	<6
Renal Creatinine, mg/dL (μmol/L) Diuresis, mL/day	<1.2 (110)	1.2–1.9 (110–170)	2–3.4 (171–299)	3.5–4.9 (300–440) <500	≥5 (440) <200

Abbreviations: FiO_2_, fraction of inspired oxygen; MAP, mean arterial pressure; PaO_2_, partial pressure of oxygen.

**Table 2:** Immune modulating strategies in critically ill septic patients.

Immune Modulating Strategies in Sepsis
Pharmacological - Interferon-γ - Granulocyte-macrophage colony-stimulating factor - Interleukin 7 - Anti-C5a - Recombinant human soluble thrombomodulin - Recombinant human-activated protein C - Intravenous Immunoglobulin - Glucocorticoids - Neutrophil elastase inhibitors - Programmed cell death protein 1/programmed death ligand - Heme oxygenase inducers
Extracorporeal blood purification therapies 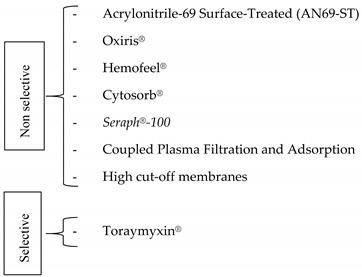

## Data Availability

Not applicable.
